# Can a Data-Driven Measure of Neuroanatomic Dementia Risk be Considered a Measure of Brain Aging?

**DOI:** 10.1093/geroni/igab046.3470

**Published:** 2021-12-17

**Authors:** Ramon Casanova, Andrea Anderson, Jamie Justice, Gwen Windham, Rebecca Gottesman, Thomas Mosley, Lynne Wagenknecht, Stephen Kritchevsky

**Affiliations:** 1 Wake Forest School of Medicine, Winston-Salem, North Carolina, United States; 2 Wake Forest School of Medicine, Wake Forest School of Medicine, North Carolina, United States; 3 University of Mississippi Medical Center, Jackson, Mississippi, United States; 4 NIH, Bethesda, Maryland, United States; 5 The University of Mississippi Medical Center, Jackson, Mississippi, United States; 6 Wake Forest School of Medicine, Winston Salem, North Carolina, United States

## Abstract

There is an increasing interest in identifying aging-related factors which may be permissive of Alzheimer’s Disease (AD) emergence. We previously used machine learning to derive an index of neuroanatomic risk of dementia called AD pattern similarity (AD-PS) score using MRIs obtained in the Atherosclerosis Risk in Communities (ARIC) study. Here, we investigate the potential of the AD-PS scores as a brain-focused measure of biologic age. Among 1970 ARIC participants with MRI collected at ARIC Visit 5, we related AD-PS scores to three measures of aging: mortality (n=356) over 8 years of follow-up; an a priori panel of 32 proteins related to aging (N=1647); and a deficit accumulation index (DAI) based on 38 health-related measures. We found lower AD-PS scores associated with significantly lower mortality (HR=0.58, CI-95%, [0.45 - 0.75], p < 0.001) after adjusting for age, race, smoking and hypertension. Among the 32 proteins, nine were significantly associated to AD-PS scores (p < 0.05) with 4 remaining significant adjusting for multiple comparisons (Growth/differentiation factor 15, Tumor necrosis factor receptor superfamily member 1A and 1B and Collagen alpha-1(XVIII) chain). Finally, in a linear regression model after adjusting for age, race, sex, hypertension and smoking, AD-PS scores were associated with the DAI (p < 0.001). The consistent patterns of associations suggest that a data-driven measure of AD neuroanatomic risk may be capturing aspects of biologic age in older adults.

